# The Effects of Aerobic Exercise Training on Cerebrovascular and Cognitive Function in Sedentary, Obese, Older Adults

**DOI:** 10.3389/fnagi.2022.892343

**Published:** 2022-05-18

**Authors:** Edward S. Bliss, Rachel H. X. Wong, Peter R. C. Howe, Dean E. Mills

**Affiliations:** ^1^Respiratory and Exercise Physiology Research Group, School of Health and Medical Sciences, University of Southern Queensland, Ipswich, QLD, Australia; ^2^Centre for Health Research, Institute for Resilient Regions, University of Southern Queensland, Ipswich, QLD, Australia; ^3^Clinical Nutrition Research Centre, School of Biomedical Sciences and Pharmacy, University of Newcastle, Callaghan, NSW, Australia; ^4^Allied Health and Human Performance, University of South Australia, Adelaide, SA, Australia

**Keywords:** aerobic exercise training, cerebrovascular function, cognition, aging, obesity

## Abstract

Cerebrovascular function and cognition decline with age and are further exacerbated by obesity and physical inactivity. This decline may be offset by aerobic exercise training (AT). We investigated the effects of 16 weeks AT on cerebrovascular and cognitive function in sedentary, obese, older adults. Twenty-eight participants were randomly allocated to AT or a control group. Before and after the intervention, transcranial Doppler ultrasonography was used to measure the cerebrovascular responsiveness (CVR) to physiological (hypercapnia, 5% carbon dioxide) and cognitive stimuli. AT increased the CVR to hypercapnia (98.5 ± 38.4% vs. 58.0 ± 42.0%, *P* = 0.021), CVR to cognitive stimuli (25.9 ± 6.1% vs. 16.4 ± 5.4%, *P* < 0.001) and total composite cognitive score (111 ± 14 vs. 104 ± 14, *P* = 0.004) compared with the control group. A very strong relationship was observed between the number of exercise sessions completed and CVR to cognitive stimuli (*r* = 0.878, *P* < 0.001), but not for CVR to hypercapnia (*r* = 0.246, *P* = 0.397) or total composite cognitive score (*r* = 0.213, *P* = 0.465). Cerebrovascular function and cognition improved following 16 weeks of AT and a dose-response relationship exists between the amount of exercise sessions performed and CVR to cognitive stimuli.

## Introduction

Aging is associated with the development of cognitive decline which may be preceded by reduced cerebrovascular function and unfavorable neuroanatomical changes (Bangen et al., 2014; Toth et al., 2017). There is also increased chronic low-grade systemic inflammation and reactive oxygen species production with aging, which promotes endothelial dysfunction and reduced cerebrovascular function and cognition (Pikula et al., 2009; Toth et al., 2017; Chang et al., 2018; Rossman et al., 2018; Bliss et al., 2021). Both cerebrovascular function and cognition are exacerbated in sedentary and obese individuals because physical inactivity and obesity promote oxidative stress, which increases the chronic low-grade inflammation, resulting in uncoupling of endothelial nitric oxide synthase which further promotes oxidative stress and inflammation leading to endothelial dysfunction (Pikula et al., 2009; Toth et al., 2017; Chang et al., 2018; Rossman et al., 2018; Bliss et al., 2021). Hence, sedentary behavior and obesity further impair cerebrovascular function, due to a reduction in capillary density and increased arterial stiffness (i.e., reduced blood flow and vasoreactivity due to decreased endothelial function) (Woods et al., 2011; Toda, 2012; Bangen et al., 2014; Toth et al., 2017). These changes diminish the metabolic capacity of the brain, as the brain is no longer supplied as efficiently as it once was with essential nutrients and oxygen and its metabolic waste is no longer removed as efficiently as in earlier adulthood. If cerebrovascular function is further reduced, the development of cerebral pathologies, cerebral dysfunction and, eventually, a neurodegenerative disease, such as dementia, can ensue as severely reduced cerebrovascular function is associated neurodegenerative changes, including tauopathies and β-amyloid deposition (Kearney-Schwartz et al., 2009; Smith and Greenberg, 2009; Bliss et al., 2021).

The population of older adults who are sedentary and obese is growing and this can lead to the development of comorbidities, including dementia (Beydoun et al., 2008; Taylor, 2014; Brown et al., 2017). The prevalence of dementia is expected to treble over the next 30 years to 150 million people globally (AIHW, 2012; Nichols et al., 2019). Thus, it is important to find and implement cost-effective and evidence-based strategies that promote improvements in cerebrovascular function and cognition which can slow or prevent the normal age-related decline and dementia. One such intervention is aerobic exercise training (AT), which has been shown to maintain cerebral perfusion and cognitive capacity in healthy aging and potentially reduce the risk of individuals developing dementia (Rogers et al., 1990; Ainslie et al., 2008; Bailey et al., 2013).

Previous research has demonstrated that moderate intensity AT interventions between 12 and 24 weeks improve cognition in middle-aged to older adults who were sedentary (Lautenschlager et al., 2008; Erickson et al., 2011; Anderson-Hanley et al., 2012; Chapman et al., 2013; Guadagni et al., 2020), suffer from mild cognitive impairment (Baker et al., 2010), or had a diagnosis of a dementia (Hoffmann et al., 2016; Sobol et al., 2016). This in contrast to the findings of a recent systematic review, which indicated that AT may not improve cognition in older adults who were both health and cognitively healthy (Young et al., 2015). Thus suggesting more research is needed in the area and that healthy older adults may be less sensitive to exercise than those who are not. Improvements in cerebral perfusion have also been reported in sedentary older adults who participated in 12 weeks of moderate intensity AT (Chapman et al., 2013; Maass et al., 2014) and in older adults with coronary artery disease following 24 weeks of AT (Anazodo et al., 2016). Cerebral pulsatility index (CPI), which determines arterial resistance and compliance of a vessel’s ability to stretch and recoil following left ventricular ejection, provides an estimation of arterial stiffness. CPI increases as compliance decreases and resistance increases, thus reflecting structural changes in the arterial walls (i.e., less elasticity) and reduced ability to adequately perfuse the area in which the vessel is supplying (Afkhami et al., 2021). CPI has been reported to decrease in patients with amnestic mild cognitive impairment who participated in 12 months AT (Tomoto et al., 2021) and following 6 months of moderate intensity AT, the cerebrovascular responsiveness (CVR) to hypercapnia increased in older, sedentary adults (Vicente-Campos et al., 2012; Kleinloog et al., 2019; Guadagni et al., 2020) and stroke patients (Ivey et al., 2011).

There are several important limitations to these studies. Firstly, the effects of AT on both cerebrovascular function and cognition are not as well defined particularly in older obese and sedentary adults who are at greater risk of developing impaired cognitive function (Beydoun et al., 2008; Pikula et al., 2009; Woods et al., 2011; Toda, 2012; Bangen et al., 2014; Toth et al., 2017; Chang et al., 2018; Rossman et al., 2018; Bliss et al., 2021). Secondly, studies have not typically measured cerebrovascular and cognitive function together. Since these functions are interrelated and contribute to overall brain health, both should be measured together to determine the effect these combined parameters exert (Bliss et al., 2021). Thirdly, no study has performed measurements of CVR to cognitive stimuli (i.e., neurovascular coupling, NVC). This is of importance because CVR to both physiological and cognitive stimuli are imperative to maintaining cerebral function because both reflect the ability of the microvasculature to maintain cerebral autoregulation and NVC (Duchemin et al., 2012; Toth et al., 2017; Miller et al., 2018). The measurement of both parameters in conjunction with cognition is essential to holistically determine the effects of AT on overall brain health. Finally, few of the studies cited have deviated from the recommended physical activity guideline of 150–300 min of moderate intensity exercise. Many older adults are not meeting these guidelines and are not participating in exercise (AIHW, 2018a; Piercy et al., 2018; Guadagni et al., 2020). The evaluation of the dose response relationship between AT and cerebrovascular and cognition function is important, as we currently do not know how much AT is required to elicit improvements in cerebrovascular and cognitive function in older adults. It may be that some exercise is better than none at delaying or preventing further cerebrovascular and cognitive decline. This may also encourage sedentary adults to exercise with a future goal of reaching the recommended physical activity guidelines.

Accordingly, the aim of the study was to evaluate the effects of 16 weeks AT performed for 2–4 days per week on cerebrovascular and cognitive function in sedentary, obese, older adults. We hypothesized that compared with control participants: (1) AT would improve both cognition and cerebrovascular function determined by the CVR to physiological and cognitive stimuli; (2) the greater the dose of AT, the greater the improvements in cerebrovascular and cognitive function.

## Materials and Methods

### Participants

Participants were recruited from the Ipswich Region, Australia between April 2019 and March 2021 *via* an approved media campaign that incorporated physical advertisement *via* media releases (flyers and newspaper articles), social media and a radio interview. Inclusion criteria were: age between 50 and 80 years; were physically inactive based on classed as physically inactive based on the physical activity guidelines of 150 min of moderate-vigorous intensity aerobic exercise per week (AIHW, 2018b); and had a body mass index (BMI) of > 25 kg/m^2^. The exclusion criteria were: aged under 50 years or over 80 years; participation in regular physical activity and meeting the physical activity guidelines of 150 min of moderate-vigorous intensity aerobic exercise per week (AIHW, 2018b); body mass index (BMI) of < 25 kg/m^2^; current smoker; blood pressure ≥ 160/100 mmHg; prescribed insulin, hormone-replacement therapy, or oral anticoagulants; had significant history of cardiovascular, cerebrovascular, kidney, liver disease or cancer; and had a diagnosis of cognitive impairment and/or a neurodegenerative disease. Participants were only included in the study if they were on a stable medication treatment plan that did not contradict the exclusion criteria. The Yale Physical Activity Survey (Dipietro et al., 1993), the Exercise and Sports Science Australia Adult Pre-exercise Screening System (Norton and Norton, 2011), and a customized health and wellbeing screen were used to determine whether participants met the inclusion and/or exclusion criteria, as well as their physical activity status and exercise behaviors. All study procedures were approved by the University of Southern Queensland Research Ethics Committee (H19REA007), which adheres to the Declaration of Helsinki. The study was registered with the Australian and New Zealand Clinical Trial Registry (ACTRN12619000988156). Participants provided written, informed consent.

### Experimental Design

The study adopted a randomized control design. The intervention lasted 16 weeks. Before and approximately 1 week following the completion of the intervention, participants visited the laboratory on two occasions, at a similar time of day, separated by a minimum of 24 h and a maximum of 7 days. Participants fasted and abstained from coffee, tea and other stimulants for 2 h before visit 1 and 8–12 h before visit 2. They were also requested to refrain from moderate-vigorous intensity exercise for 24 h before each visit and to take their daily supplements and medication after each visit was completed. During visit 1, participants undertook anthropometric, cardiovascular, exercise performance, strength, cerebrovascular and cognitive measurements. During visit 2, participants undertook body composition measurements, blood collection and the Profile of Moods State questionnaire. The Profile of Mood States questionnaire calculated mood disturbance by adding the scores of the negative mood state scales (i.e., anger-hostility, confusion-bewilderment, depression-dejection, fatigue inertia, tension-anxiety) and subtracting the positive mood state scale (i.e., vigor-activity) (Heuchert and McNair, 2012). Lower values in the negative mood states and total mood disturbance indicated better mood, while higher values in the positive mood state portion of the questionnaire are associated with positive mood. Between visits 1 and 2, participants were asked to complete a nutritional questionnaire (Automated Self-Administered 24 h Dietary Assessment Tool; National Institute of Health, Bethesda, MA, United States) to estimate energy intake over a typical 24 h period (Pannucci et al., 2018).

### Exercise Intervention

Participants were then randomly allocated to one of two arms of the study using Altman’s minimization method (prioritizing BMI and sex) to ensure that the groups were balanced (Altman and Bland, 2005). The two arms of the study included a control group, which did not participate in any exercise training, and an exercise group. Participants allocated to the exercise group were asked to participate in AT for between 2–4 days per week (i.e., they could participate in either two, three, or four sessions per week). These sessions were conducted as group classes supervised by a clinical exercise physiologist. Table 1 provides an overview of the exercise session content. All sessions lasted for approximately 40–45 min and incorporated both a 5 min warm-up and cool-down. The body of the sessions was performed at a rating of perceived exertion for leg discomfort of 5–8 on the Borg (CR-10) scale, which indicated that the exercise was being performed at a moderate (5–6) or high intensity (7–8) (Borg, 1998). Additionally, the participants were asked to perform these exercise sessions at a higher intensity at approximately every 4 weeks if possible (Table 1). The circuit comprised functional exercises including wall press-ups, marching, step-ups, sit-to-stands, squats and basic resistance exercises. Compliance was measured by a roll call at the start of every exercise session to determine participation. Participants were contacted by either by phone, email, or in person each week for the first 2 weeks of the study, then fortnightly until 8 weeks at which time they were contacted every 4 weeks to ensure protocol familiarity, personal reassurance and motivation and as a check of their general wellbeing. This practice can assist in ensuring participant compliance (Lautenschlager et al., 2008). An attendance rate of 40% of all of the available exercise sessions at the end of the 16 weeks was considered compliant (Rejeski et al., 1997; Claxton et al., 2001; Lautenschlager et al., 2008).

**TABLE 1:** Composition of the exercise sessions, including session content, intensity and rating of perceived exertion.

Week/s	Day/s	Session	Intensity	Rating of perceived exertion (0–10)
1–2	Monday/Friday	30 Min walking	Moderate	5
	Tuesday/Thursday	15 Min walking 15 min circuit	Moderate Moderate-to-vigorous	5 6–7
3–5	Monday	35 min walking	Moderate	5–6
	Tuesday/Thursday	15 Min walking, cycling, arm ergometry, rowing 15 min circuit	Moderate Vigorous	5–6 7–8
	Friday	15 Min walking 3 × 6 min circuits	Moderate	5–6
6–8	Monday/Friday	15 Min walking, cycling, arm ergometry, rowing 3 × 5 min circuits	Moderate Moderate	6 6
	Tuesday/Thursday	15 Min walking, cycling, arm ergometry, rowing 15 min circuit	Moderate Vigorous	6 7–8
9–12	Monday/Friday	15 Min walking, cycling, arm ergometry, rowing 3 × 5 min circuits	Moderate Moderate	6 6
	Tuesday/Thursday	15 Min walking, cycling, arm ergometry, rowing 15 min circuit	Moderate Vigorous	6 7–8
13–14	Monday/Friday	5 Min walking, cycling, arm ergometry, rowing 7 × 4 min circuits	Moderate Moderate-to-vigorous	6 7
	Tuesday/Thursday	10 Min walking, cycling, arm ergometry, rowing 20 min circuit	Moderate Vigorous	6 8
15–16	Monday/Friday	5 Min walking, cycling, arm ergometry, rowing 7 × 4 min circuits	Moderate Moderate-to-vigorous	6 7
	Tuesday/Thursday	5 Min walking, cycling, arm ergometry, rowing 2 × 15 min circuits	Moderate Vigorous	6 8

### Basal Cerebral Hemodynamics

Transcranial Doppler ultrasonography (TCD; DopplerBox X; Compumedics DWL, Singen, Germany) was used to measure basal cerebrovascular hemodynamics, including minimum, maximum and mean values for both CBF_*V*_ and cerebral pulsatility, as well as CVR in response to hypercapnia and cognitive stimuli following at least 10 min of quiet rest in a seated position (Edmonds et al., 2011; Barbour et al., 2017; Evans et al., 2017). Participants were seated and fitted with a headpiece which housed two 2-MHz TCD ultrasound probes that were fixed and aligned bilaterally to the left and right cranial temporal bone windows to insonate the middle cerebral arteries (MCA) at a depth of approximately 40–65 mm through the transtemporal window using standardized techniques as previously described (Edmonds et al., 2011; Willie et al., 2011). Once a suitable blood flow signal was obtained, participants were asked to sit quietly while basal measurements were recorded for 30 s. If the MCA could not be insonated, the participant was excluded from the study.

### Cerebrovascular Responsiveness to Hypercapnia

Participants were subsequently challenged with a hypercapnic stimulus for 3 min and monitored for another 1 min following removal. This process was performed in duplicate following a 5 min rest period (whilst participants breathed in room air) to ensure mean velocity returned to baseline values (Barbour et al., 2017; Evans et al., 2017). Participants breathed through a two-way non-rebreathing valve (model 2730, Hans Rudolph, Kansas City, MO, United States) whilst wearing a nose-clip. The inspiratory port of the two-way valve was connected to 1 m of wide bore tubing distal to a 100 L Douglas bag which contained carbogen gas (5% carbon dioxide and 95% oxygen; Carbogen 5; BOC, Toowoomba, Australia). Flow was measured from the expiratory port of the two-way valve using a pneumotachograph (MLT 300L; AD Instruments, Bella Vista, Australia) which was calibrated with a 3 L syringe prior to the commencement of each test. Volume was obtained by numerical integration of the flow signal. End-tidal partial pressures of carbon dioxide (P_*ET*_CO_2_) were sampled from the expiratory port of the two-way valve connected to a gas analyzer (ADI ML206; AD Instruments, Bella Vista, Australia) that was calibrated across the physiological range with known gas concentrations (BOC, Toowoomba, Australia). Flow and P_*ET*_CO_2_ measurements were sampled at 200 Hz using a 4-channel Powerlab analog-to-digital converter (AD Instruments, Bella Vista, Australia) interfaced with a computer and displayed in real time during testing. Data were stored for subsequent offline analysis using LabChart software (version 7.2, AD Instruments, Bella Vista, Australia).

### Cognitive Function and Cerebrovascular Responsiveness to Cognitive Stimuli

Cognitive tests included the Trail Making Task Parts A and B which assessed central executive function, Spatial Span Test (visuospatial short-term working memory) and a National Institute of Health (NIH) Toolbox, which is a battery of cognitive examinations (Strauss et al., 2006; Evans et al., 2017). The NIH Toolbox is comprised of the Dimensional Change Card Sort Test (cognitive flexibility and attention), Picture Vocabulary Test (language and crystallized cognition), List Sorting Working Memory Test (working memory), Oral Reading Recognition Test (language and crystallized cognition), Flanker Inhibitory Control and Attention Test (attention and inhibitory control), Picture Sequence Memory Test (episodic memory), Pattern Comparison Processing Speed Test (processing speed) (Slotkin et al., 2012; Heaton et al., 2014). An age adjusted total composite cognitive function score was also derived from the above (Heaton et al., 2014). All NIH Toolbox test scores were automatically computed within the program to control for examiner bias. The outputs for all tests were normalized based on the demographics entered into the program (age, education level, familial education history, sex, ethnicity, and occupation). A full description of how these tests are administered, how these scores are calculated and the validation of these tests and scores have been previously described in detail (Slotkin et al., 2012; Heaton et al., 2014; Weintraub et al., 2014). All tests excluding the Trail Making Task were delivered using an iPad (6th generation, Apple Inc., Cupertino, CA, United States). The CVR to cognitive stimuli was assessed during each cognitive task and 30 s of baseline data was recorded before the start of each cognitive task.

### Data Capture and Processing for Cerebrovascular Responsiveness

Beat-to-beat measurements of CBF_*V*_ were recorded from the MCA onto software (QL Reader; Compumedics DWL, Singen, Germany) sampling at 100 Hz and were stored for subsequent offline analysis. If a bilateral signal was not obtained, then analysis took place with only the side that was able to be obtained. These data were then normalized and analyzed using Curve Expert Professional software (Hyams Development, Chattanooga, TE, United States) to determine peak CBF_*V*_, resting CBF_*V*_ and resting cerebral pulsatility index (CPI). CVR and CPI were calculated based on Equations (1) and (2) from previous work (Bakker et al., 2004; Wong R. H. X. et al., 2016; Harris et al., 2018).


(1) $$\mathrm{CPI}=\,\frac{\mathrm{peak}\,\mathrm{systolic}\,\mathrm{CBFv}-\mathrm{end}\,\mathrm{diastolic}\,\mathrm{CBFv}}{\mathrm{mean}\,\mathrm{CBFv}\,\mathrm{during}\,a\,\mathrm{cardiac}\,\mathrm{cycle}}$$

(2) $$\mathrm{CVR}\left(\%\right)=\frac{\left(\mathrm{peak}\,\mathrm{CBFv}-\mathrm{resting}\,\mathrm{CBFv}\right)}{\mathrm{resting}\,\mathrm{CBFv}}\times 100÷\mathrm{resting}\,\mathrm{CPI}$$

### Anthropometrics and Body Composition

Participants were instructed to wear light clothing prior to testing and subsequently asked to remove their shoes for measurements. Body mass was measured to the nearest 100 g using an electronic scale (Tanita Ultimate Scale, 2000; Tokyo, Japan) and waist and hip circumferences was recorded to the nearest 1 cm using a standard tape measure as previously described (Welborn et al., 2003). Height was recorded to the nearest 1 cm using a wall-mounted telescopic stadiometer (Seca220; Vogel and Halke, Hamburg, Germany). Height, body mass and waist and hip circumference measurements were measured in duplicate and the mean of the two measurements were analyzed. BMI and a waist to hip ratio were calculated as previously described (Keys et al., 1972; Welborn et al., 2003). Dual-energy X-ray absorptiometry was used to obtain the following measures of whole body composition: lean mass, body fat percentage, bone mineral content and density (Luna Corp. Prodigy Advance Model GE; Madison, WI, United States).

### Cardiovascular Function

Systolic and diastolic blood pressure, mean arterial pressure, heart rate and arterial elasticity were measured non-invasively using a HDI/Pulsewave™ CR-2000 Research Cardiovascular Profiling System (Hypertension Diagnostics, Eagan, MN, United States) (Prisant et al., 2002). Participants rested in a seated position for 10 min prior to measurements. Four consecutive readings were recorded approximately 5 min apart by an automated oscillometer, using an appropriately size blood pressure cuff over the left brachial artery, to assess blood pressure and a tonometer, placed over the right radial artery, to assess heart rate and estimate arterial elasticity, cardiac output and cardiac index by pulse wave analysis (Prisant et al., 2002; Barbour et al., 2017). The first reading was discarded and the mean of the three subsequent readings was used for analysis.

### Biochemical Analyses

Approximately 20 ml of venous blood was sampled using a suitable method (i.e., either evacuated tube system or winged-infusion set for difficult collections) from the veins of the antecubital fossa into a thrombin-based clot activator serum separator tubes (BD, Macquarie Park, NSW, Australia). Following collection, blood was left to stand for 30 min at 18–25°C prior to centrifugation at 1,300 g and 18°C for 10 min, as outlined by the tube manufacturer and the testing laboratory (BD, 2019; QML, 2019). Following centrifugation, blood was separated as serum and analyzed for the general chemistry profile and high-sensitivity C-reactive protein (hs-CRP) on a Siemens ADVIA^®^ Labcell^®^ (Siemens Healthcare, Bayswater, VIC, Australia), which utilizes spectrophotometric (enzymes, metabolites, proteins, lipids), turbidimetric (hs-CRP) and potentiometric (electrolytes) techniques (Healthineers, 2019).

### Exercise Performance and Handgrip Strength

Exercise performance was assessed using a 6 min walk test (6MWT) according to published guidelines (ATS, 2002). Handgrip strength was determined using hand dynamometry as previously described (Hillman et al., 2005). Participants were permitted three attempts with both their dominant and non-dominant hands. The first reading for each hand was discarded and was used as a familiarization and the second and third readings for each hand were averaged for each hand and were used for analysis. Both the 6MWT and handgrip strength were used to provide an estimate of endurance exercise capacity and whole-body strength (ATS, 2002; Hillman et al., 2005).

### Statistical Analysis

Statistical analyses were performed using SPSS for Windows (IBM, Chicago, United States). An initial power calculation was performed on the basis of previous research that has investigated the differences in CVR between participants who had undergone an intervention study (Wong R. et al., 2016; Wong R. H. X. et al., 2016; Barbour et al., 2017; Evans et al., 2017). It indicated that 32 participants would give 80% power to detect a significant (*p* < 0.05) 5% increase in the CVR to hypercapnia following exercise training. This was based on a 10% standard deviation observed in previous studies and a medium sized effect (Wong R. et al., 2016; Wong R. H. X. et al., 2016; Barbour et al., 2017; Evans et al., 2017). Recruitment was limited to 28 participants due to the impacts of COVID-19 and the restrictions placed on gathering, research and travel by the Queensland Government. Normality of data was assessed using a Shapiro-Wilk test. Comparisons between groups for anthropometric, body composition, cardiovascular, cognitive, exercise performance, strength and biochemical measures were determined using an independent *t*-test or a Mann-Whitney *U*-test for parametric and non-parametric data, respectively. A two-way analysis of variance (ANOVA) was used to determine the effects of “treatment” (exercise vs. control) and “intervention” (week 0 vs. week 16). A three-way ANOVA was used to determine the effects of “treatment,” “intervention” and “time” (baseline vs. peak) for the CVR to hypercapnia and cognitive stimuli measures. Significant interaction effects were followed by planned pairwise comparisons between groups using the Bonferroni method. Pearson product-moment correlation coefficients were calculated to assess the relationship between the number of exercise sessions completed and the relative percentage change from week 0 to week 16 for the CVR to hypercapnia, total composite CVR to cognitive stimuli and total composite cognitive score in the exercise group (Schober et al., 2018). The relative percentage change for each of these variables were determined by the following formula:


(3) $$\mathrm{relative}\,\mathrm{percentage}\,\mathrm{increase}=\frac{\left(\text{week}\,16\,\mathrm{value}-\text{week}\,0\,\mathrm{value}\right)}{\text{week}\,0\,\mathrm{value}}\times 100$$

Statistical significance was set at *P* < 0.05. Effect sizes were determined using Cohen’s *d* using the following thresholds: ≤ 0.19 = trivial, ≥ 0.2 ≤ 0.49 = small, ≥ 0.5 ≤ 0.79 = medium, and ≥ 0.8 = large (Cohen, 1988). Results are presented as means ± SD.

## Results

### Participant Characteristics

**FIGURE 1:**
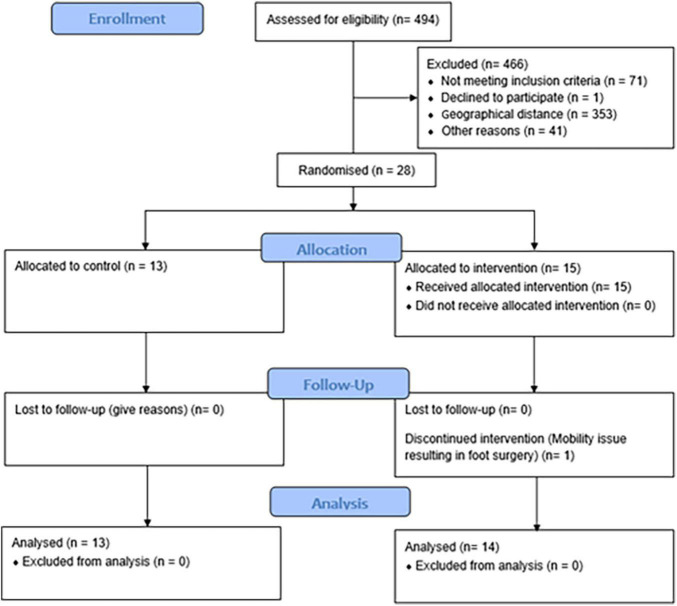
CONSORT participant flow diagram.

Figure 1 shows the CONSORT participant flow diagram. Twenty-seven participants were included in the final data and statistical analysis. Thirteen participants were in the control arm of the study. Fourteen participants completed the exercise intervention and compliance was good with 40 ± 3 (63 ± 5%) exercise sessions completed.

Baseline characteristics are shown in Table 2. No differences were observed between the groups except for the number of flights of stairs climbed daily which was higher in the control compared to the exercise group with a medium effect size (*d* = 0.68). Baseline general biochemistry profiles and hs-CRP are shown in Table 3. There were no differences between the groups. The participants were obese, had low-grade inflammation and met the International Diabetes Federation criteria of the metabolic syndrome (Alberti et al., 2005).

**TABLE 2:** Baseline demographics, anthropometrics, nutritional intake and physical activity levels for the control and exercise groups.

Variable	Control (*n* = 13)	Exercise (*n* = 14)	*P*-value
***Demographics***			
Age (years)	66 ± 9	67 ± 7	0.553
Sex (male/female)	2/11	4/10	0.618
Education (years)	16 ± 4	14 ± 3	0.440
***Anthropometrics***			
Body mass (kg)	93.5 ± 23	88.7 ± 11	0.928
Height (m)	1.64 ± 0.08	1.68 ± 0.09	0.208
Body mass index (kg/m^2^)	34.6 ± 6.55	31.4 ± 1.99	0.339
Hip circumference (cm)	126 ± 15	117 ± 5	0.118
Waist circumference (cm)	114 ± 15	107 ± 8	0.217
Hip-to-waist ratio	0.91 ± 0.07	0.91 ± 0.08	0.730
***Nutritional intake***			
Total energy intake (kcal)	2,375 ± 769	2,688 ± 1,035	0.393
***Physical activity levels***			
Energy expenditure (kcal/min)	70 ± 34	94 ± 73	0.560
Vigorous activity index	5 ± 5	1 ± 2	0.131
Leisurely walking index	5 ± 5	8 ± 10	0.527
Moving index	7 ± 3	8 ± 4	0.705
Standing index	5 ± 1	6 ± 4	0494
Sitting index	4 ± 2	4 ± 2	0.560
Flights of stairs climbed per day	2 ± 3	1 ± 2	**0.036**
Seasonal adjustment score	1 ± 0	1 ± 0	0.274

*Values are means ± SD.*

*Bold values represent significant differences.*

**TABLE 3:** Baseline biochemical analyses for the control and exercise groups.

Variable	Control (*n* = 11)	Exercise (*n* = 14)	*P*-value
Glucose (mmol/L)	5.7 ± 1.4	5.6 ± 1.0	0.683
Urea (mmol/L)	6.1 ± 1.5	5.9 ± 1.1	0.610
Creatinine (mmol/L)	74 ± 21	67 ± 10	0.959
Estimated glomerular filtration rate (ml/min)	80 ± 14	84 ± 10	0.384
Total bilirubin (μmol/L)	9 ± 4	9 ± 3	0.574
Alkaline phosphatase (U/L)	92 ± 26	75 ± 27	0.123
Gamma-glutamyl transferase (U/L)	35 ± 25	29 ± 16	0.760
Alanine aminotransferase (U/L)	37 ± 20	30 ± 10	0.443
Aspartate aminotransferase (U/L)	35 ± 15	27 ± 5	0.384
Lactate dehydrogenase (U/L)	234 ± 69	202 ± 30	0.474
Total protein (g/L)	70 ± 3	69 ± 4	0.555
Albumin (g/L)	40 ± 3	42 ± 2	0.164
Globulins (g/L)	30 ± 5	27 ± 3	0.101
Total cholesterol (mmol/L)	5.3 ± 1.0	5.6 ± 1.0	0.330
Triglycerides (mmol/L)	1.4 ± 0.8	1.4 ± 0.6	0.931
High-density lipoprotein (mmol/L)	1.47 ± 0.22	1.45 ± 0.35	0.827
Low-density lipoprotein (mmol/L)	2.71 ± 0.87	3.18 ± 0.83	0.186
Total cholesterol-to-high-density lipoprotein ratio	3.7 ± 0.9	4.1 ± 0.8	0.292
High-sensitivity C-reactive protein (mg/L)	4.8 ± 2.8	3.0 ± 2.6	0.066

*Values are means ± SD.*

### Body Composition, Grip Strength, Exercise Performance, Cardiovascular Function

Body composition, grip strength, exercise performance and cardiovascular function measurements are shown in Table 4. The 6MWT distance at week 0 was higher in the exercise compared to the control group with a medium effect size (main effect of treatment, *d* = 0.74). Total lean mass (*d* = 0.18), 6MWT distance, and large arterial compliance (*d* = 0.08) increased in both groups (main effect of intervention), while total body fat percentage (*d* = 0.69), systolic blood pressure (*d* = 0.19), mean arterial pressure (*d* = 0.04) and total vascular impedance (*d* = 0.01) decreased in both groups (main effect of intervention). There were no significant treatment x intervention interaction effects in any of the parameters, although the 6MWT distance (*d* = 1.33) and total bone mineral content (*d* = 0.31) were approaching significance.

**TABLE 4:** Body composition, grip strength, exercise performance and cardiovascular function at week 0 and week 16 for the control and exercise groups.

Variable	Control (*n* = 13)		Exercise (*n* = 14)		*P*-value		
	Week 0	Week 16	Week 0	Week 16	Treatment	Intervention	Treatment × intervention
***Body composition***							
Total lean mass (kg)	45.6 ± 8.6	46.8 ± 9.6	48.2 ± 10.4	48.7 ± 10.3	0.551	**0.029**	0.299
Total body fat (%)	48.4 ± 6.7	47.1 ± 7.4	43.5 ± 5.6	42.7 ± 5.5	0.064	**<0.001**	0.353
Total bone mineral density (g/cm^2^)	1.24 ± 0.13	1.24 ± 0.13	1.22 ± 0.14	1.22 ± 0.14	0.673	0.762	0.827
Total bone mineral content (g/cm)	2,478 ± 458	2,439 ± 476	2,598 ± 557	2,602 ± 565	0.484	0.113	0.052
***Grip strength***							
Dominant hand (kg)	27.0 ± 7.8	27 ± 7.6	28.7 ± 9.7	31.3 ± 10.3	0.332	0.364	0.324
Non-dominant hand (kg)	26.1 ± 6.3	25.8 ± 7.0	26.8 ± 8.7	30.1 ± 9.6	0.336	0.242	0.147
***Exercise performance***							
6-Min walk test distance (m)	461 ± 73	469 ± 92	511 ± 61	576 ± 66	**0.004**	**0.015**	0.058
***Cardiovascular function***							
Heart rate (beats/min)	76 ± 12	75 ± 10	72 ± 6	71 ± 10	0.213	0.719	0.810
**Systolic blood pressure (mmHg)**	144 ± 15	133 ± 14	139 ± 9	130 ± 10	0.347	**<0.001**	0.473
Diastolic blood pressure (mmHg)	78 ± 8	73 ± 10	76 ± 6	72 ± 5	0.401	0.055	0.714
Mean arterial pressure (mmHg)	104 ± 9	95 ± 10	99 ± 11	95 ± 8	0.336	**0.012**	0.183
Large arterial compliance (ml/mmHg × 10)	9.3 ± 3.4	11.5 ± 3.9	9.7 ± 3.4	11.8 ± 4.1	0.735	**0.007**	0.885
Small arterial compliance (ml/mmHg × 10)	3.9 ± 1.8	4.5 ± 2.0	4.5 ± 2.0	4.8 ± 3.3	0.501	0.291	0.532
Systemic vascular resistance (dyne/sec/cm^–^*^s^*)	1,663 ± 316	1,534 ± 263	1,630 ± 261	1,543 ± 229	0.836	0.125	0.708
Total vascular impedance (dyne/sec/cm^–^*^s^*)	202 ± 59	161 ± 48	178 ± 39	161 ± 44	0.431	**0.010**	0.218

*Values are means ± SD.*

*Bold values represent significant differences.*

### Cerebrovascular Responsiveness to Hypercapnia

**FIGURE 2:**
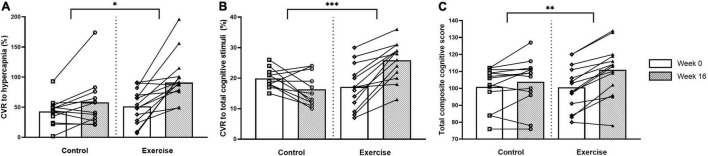
Cerebrovascular responsiveness (CVR) to hypercapnia **(A)**, CVR to total composite of cognitive stimuli **(B)** and total composite cognitive score **(C)** at weeks 0 and 16 for the control and exercise groups. Significant difference between groups * (P < 0.05), ^**^ (P < 0.005), ^***^ (P < 0.001).

The CVR to hypercapnia and the cerebrovascular and respiratory parameters are shown in Figure 2 and Table 5. All variables measured increased during hypercapnia (main effect of time; *P* < 0.001), except for CPI which decreased (main effect of time; *P* < 0.001) and breathing frequency, which did not change. The CVR to hypercapnia increased to a greater extent in the exercise than the control group (+ 40%; treatment x intervention interaction; *P* = 0.021, *d* > 0.82). Although Week 0 CBF_*V*_ was lower in the exercise group than the control group, the increase in CBF_*V*_ during hypercapnia (treatment × intervention × time interaction) was greater following the intervention (+ 36%) in the exercise group (*d* = 0.81). CPI decreased (main effect of intervention, *P* = 0.001, *d* = 0.24) in both the exercise and control groups following the intervention but there were no interaction effects. Maximum tidal volume and minute ventilation (treatment x intervention interaction) increased to a greater extent in the exercise compared to the control group following the intervention (*d* > 0.82 and *d* = 0.28).

**TABLE 5:** Cerebrovascular and respiratory parameters at baseline and peak hypercapnia at week 0 and week 16 for the control and exercise groups.

Variable	Control (*n* = 13)				Exercise (*n* = 14)				Treatment	Intervention	Treatment × intervention	Treatment × intervention × time
	Week 0		Week 16		Week 0		Week 16					
	Baseline	Peak	Baseline	Peak	Baseline	Peak	Baseline	Peak				
***Cerebrovascular variables***												
CBF_*V*_ (cm/s)	37.4 ± 9.3	52.3 ± 15.4	41.6 ± 13.5	61.2 ± 18.2	31.0 ± 8.9	47.0 ± 14.0	33.5 ± 5.3	61.2 ± 8.7	0.147	**< 0.001**	0.116	**0.045**
CPI	1.22 ± 0.44	1.05 ± 0.50	1.00 ± 0.25	0.83 ± 0.19	1.20 ± 0.32	0.93 ± 0.18	0.95 ± 0.16	0.80 ± 0.13	0.971	**0.001**	0.319	0.350
***Respiratory variables***												
P_*ET*_CO_2_ (mmHg)	32.5 ± 5.1	37.1 ± 5.0	33.8 ± 5.2	38.6 ± 5.4	29.6 ± 3.2	35.9 ± 4.3	30.9 ± 4.1	37.2 ± 3.5	0.982	0.332	0.997	0.884
Tidal volume (L)	0.79 ± 0.29	1.13 ± 0.46	0.62 ± 0.27	0.93 ± 0.37	0.80 ± 0.28	0.98 ± 0.39	1.00 ± 0.25	1.34 ± 0.49	0.099	0.280	**0.002**	0.166
Breathing frequency (breaths/min)	16 ± 8	18 ± 8	16 ± 7	18 ± 10	15 ± 5	16 ± 7	13 ± 4	15 ± 6	0.406	0.434	0.200	0.812
Minute ventilation (L/min)	12.0 ± 4.1	16.8 ± 9.1	9.3 ± 4.1	14.8 ± 8.3	11.3 ± 4.9	14.6 ± 5.2	12.0 ± 4.9	17.0 ± 7.5	0.619	0.885	**0.035**	0.663
CBF_*V*_/P_*ET*_CO_2_ (cm/s/mmHg)	1.24 ± 0.20	1.53 ± 0.31	1.24 ± 0.35	1.59 ± 0.42	1.10 ± 0.20	1.41 ± 0.31	1.10 ± 0.26	1.65 ± 0.23	0.232	0.263	0.853	0.384

*CBF_V_, cerebral blood flow velocity; CPI, cerebral pulsatility index; P_ET_CO_2_, partial pressure of end tidal carbon dioxide.*

*Values are means ± SD.*

*Bold values represent significant differences.*

### Cognitive Function and Cerebrovascular Responsiveness to Cognitive Stimuli

Cognitive function and the CVR to cognitive stimuli are shown in Figure 2 and Table 6. There were no differences between the groups in any of the cognitive parameters at week 0. Following the intervention, the exercise group had higher overall cognitive function than the untrained group, which was demonstrated by a higher total cognitive composite score with a small effect size (3 vs. 10% improvement; treatment × intervention interaction; *P* = 0.004, *d* = 0.49). The exercise group also had increased working memory capacity compared to the control group with a large effect size following the intervention, demonstrated by the List Sorting Working Memory Test (*d* > 0.82).

**TABLE 6:** Cognitive function and cerebrovascular responsiveness to cognitive stimuli at week 0 and week16 for the control and exercise groups.

Variable	Control (*n* = 13)		Exercise (*n* = 14)		*P*-value		
	Week 0	Week 16	Week 0	Week 16	Treatment	Intervention	Treatment × intervention
***Cognitive function***							
Dimensional change card sort*	7.46 ± 1.33	7.78 ± 0.15	7.32 ± 2.00	7.58 ± 1.25	0.708	0.313	0.939
Pattern comparison processing speed*	43 ± 11	44 ± 11	47 ± 10	53 ± 13	0.164	**0.006**	0.078
Picture vocabulary test*	6.16 ± 1.86	6.06 ± 2.10	5.14 ± 2.37	5.99 ± 2.24	0.492	0.215	0.118
Flanker inhibitory control and attention*	7.80 ± 0.49	7.79 ± 0.40	8.09 ± 0.47	8.16 ± 0.43	0.062	0.443	0.365
Picture sequence memory*	–0.67 ± 0.84	–0.36 ± 0.98	–0.90 ± 0.58	–0.62 ± 0.18	0.379	**0.005**	0.886
List sorting working memory*	16 ± 2	16 ± 3	15 ± 3	19 ± 3	0.352	**< 0.001**	**<0.001**
Oral reading recognition*	4.61 ± 2.41	5.10 ± 2.59	4.16 ± 2.32	5.71 ± 2.57	0.921	**0.002**	0.080
Trail making task (Part A)							
Time (s)	36.3 ± 13.1	36.5 ± 14.1	37.0 ± 14.1	28.6 ± 12.4	0.410	0.173	0.152
Errors made	0.7 ± 0.9	0.9 ± 1.3	0.6 ± 0.9	0.1 ± 0.4	0.107	0.602	0.164
Trail making task (Part B)							
Time (s)	83.4 ± 48.9	66.6 ± 23.9	77.9 ± 29.2	67.2 ± 47.3	0.723	0.216	0.621
Errors made	2.4 ± 1.9	2.5 ± 4.6	1.5 ± 2.2	0.6 ± 0.7	0.052	0.550	0.687
Part B–Part A time difference	47.1 ± 38.8	30.0 ± 16.5	40.9 ± 20.3	38.5 ± 36.8	0.951	0.315	0.262
Spatial span test							
Time (s)	100.9 ± 20.5	104.3 ± 38.4	97.4 ± 35.9	105.3 ± 22.6	0.769	0.504	0.752
Total spans completed	5.6 ± 0.9	5.2 ± 1.6	4.8 ± 1.3	5.4 ± 0.7	0.215	0.697	0.129
Total composite cognitive score	101 ± 12	104 ± 14	101 ± 13	111 ± 14	0.508	**< 0.001**	**0.004**
***Cerebrovascular responsiveness (%)***							
Dimensional change card sort test	16.9 ± 8.9	12.1 ± 5.0	13.3 ± 7.0	20.8 ± 6.1	0.329	0.289	**0.001**
Pattern comparison processing speed test	20.0 ± 6.5	18.7 ± 10.2	18.0 ± 6.8	24.5 ± 7.1	0.517	0.179	**0.048**
Picture vocabulary test	23.1 ± 7.0	21.9 ± 11.9	20.0 ± 9.4	27.2 ± 7.9	0.797	0.192	0.083
Flanker inhibitory control and attention test	13.1 ± 8.5	14.0 ± 6.7	12.3 ± 6.1	19.2 ± 5.6	0.342	**0.026**	0.080
Picture sequence memory test	21.9 ± 7.3	17.5 ± 6.0	20.6 ± 9.2	27.2 ± 11.6	0.238	0.529	**0.010**
List sorting working memory test	21.0 ± 5.9	16.8 ± 5.7	17.8 ± 7.2	28.1 ± 8.0	0.112	**< 0.001**	**0.040**
Oral reading recognition test	19.9 ± 4.2	10.4 ± 6.5	14.0 ± 8.0	26.9 ± 10.0	0.067	0.572	**< 0.001**
Trail making task (Part A)	24.0 ± 9.2	16.6 ± 5.2	20.0 ± 9.7	25.8 ± 8.4	0.405	0.441	**< 0.001**
Trail making task (Part B)	17.4 ± 6.5	16.9 ± 5.4	19.5 ± 10.4	30.6 ± 5.5	**0.016**	0.153	**0.002**
Spatial span test	22.3 ± 4.3	16.3 ± 6.1	16.4 ± 9.3	25.8 ± 7.3	0.511	0.226	**< 0.001**
Total composite CVR to cognitive stimuli	19.9 ± 3.3	16.4 ± 5.4	19.5 ± 10.4	25.9 ± 6.1	0.114	**0.026**	**< 0.001**

*Normalized, computed and standardized automatically by NIH Toolbox, based on validated measures (Slotkin et al., 2012).

*Values are means ± SD.*

*Bold values represent significant differences.*

At week 0, there were no differences between the groups in any CVR measures except for the CVR to Part B of the Trail Making Task which was higher in the control than the exercise group with a small effect size (main effect of treatment, *d* = 0.23). Following the intervention, the exercise group had a higher total composite CVR to all cognitive stimuli (10% higher; treatment x intervention interaction; *P* < 0.001, *d* > 0.82) than the control group with a large effect size, as well as a higher CVR to all of the individual cognitive stimuli with a large effect size (*d* > 0.82), excluding the Picture Vocabulary Test and the Flanker Inhibitory Control and Attention Test (treatment × intervention interaction).

### Correlations Between Exercise Sessions Completed and Cerebrovascular Function and Cognition

**FIGURE 3:**
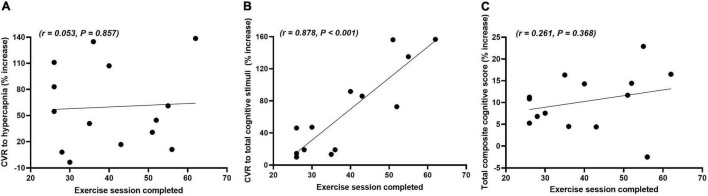
Correlations between the number of exercise sessions completed and the relative percentage change (% increase) in the cerebrovascular responsiveness (CVR) to hypercapnia **(A)**, total composite CVR to cognitive stimuli **(B)** and total composite cognitive score **(C)** from weeks 0 to 16 in the exercise group.

Figure 3 shows the correlations between the number of exercise sessions completed and the percentage increase from week 0 to week 16 for the CVR to hypercapnia, total composite CVR to cognitive stimuli and total composite cognitive score in the exercise group. There were no significant differences in the frequency of sessions in which participants chose to participate in on a weekly basis over the 16-week period (2.5 ± 0.3 sessions). There was a very strong positive correlation between the number of exercise sessions completed and the total composite CVR to cognitive stimuli. There were no significant correlations between the number of exercise sessions completed and either the CVR to hypercapnia or the total composite cognitive score.

## Discussion

### Main Findings

The aim of the study was to evaluate the effects of 16 weeks AT on cerebrovascular and cognitive function in sedentary, obese, older adults. We hypothesized that compared with control participants: (1) AT would improve both cognition and cerebrovascular function determined by the CVR to physiological and cognitive stimuli; and (2) the greater the dose of AT, the greater the improvements in cerebrovascular and cognitive function. The main findings were that AT increased the CVR to hypercapnia, CVR to cognitive stimuli and total composite cognitive score compared with the control group. A very strong relationship was observed between the number of exercise sessions completed and CVR to cognitive stimuli, but not for CVR to hypercapnia or total composite cognitive score. These results demonstrate that in sedentary, obese, older adults 16 weeks of AT can improve both improve both cognition and cerebrovascular function and there is a dose response relationship between the number of exercise sessions completed and CVR to cognitive stimuli.

### Cerebrovascular Responsiveness to Hypercapnia

We observed an increase in the CVR to hypercapnia following AT in the exercise compared with the control group. This finding is similar to others who have also reported an increased CVR to hypercapnia in older, sedentary adults (Vicente-Campos et al., 2012; Guadagni et al., 2020) and stroke patients (Ivey et al., 2011) following 6 months of moderate intensity AT. We also observed that the increase in CBF_*V*_ during hypercapnia was greater following the intervention in the exercise compared to the control group. Increased cerebral perfusion, whether measured directly or indirectly, following at least 12 weeks of AT is consistent with other studies that reported increases in either CBF or CBF_*V*_ in older adults who were previously sedentary (Vicente-Campos et al., 2012; Chapman et al., 2013; Maass et al., 2014) and those with coronary artery disease who undertook 6 months of AT (Anazodo et al., 2016). We found that CPI decreased in both the exercise and control groups following the intervention, but there were no differences between the groups. A previous study reported no change in basal CPI in middle-aged to older sedentary adults who undertook a 12 week moderate intensity AT (Akazawa et al., 2018). However, it was reported that these adults did have a decreased CPI following 30 min of acute exercise. The limitation of this study was that it was not a randomized control trial and only compared CPI changes between weeks 0 and 12. The decreased CPI reported following an acute bout of exercise may largely reflect post-exercise recovery, making it difficult to ascertain if an AT intervention can cause a sustained improvement of CPI in older adults (Akazawa et al., 2018). The participants in that study differed from ours in that they were not obese and did not have cardiometabolic disease, thus suggesting that changes in CPI in those who are sedentary, obese and older may take longer than 12–16 weeks to elicit following an AT intervention. Further studies in this area are warranted to determine if AT can reduce arterial stiffness. In any case, our results collectively indicate that 16 weeks of AT can improve cerebrovascular function. The mechanisms for this improvement may be due to an increased ability of the microvasculature to respond to local chemical changes, modify regional blood flow in response to these changes and maintain cerebral autoregulation *via* improved endothelial function and its ability to synthesize nitric oxide (NO) in response to shear stress (Serrador et al., 2000; Willie et al., 2011; Duchemin et al., 2012; Toth et al., 2017). This may translate to improvements in the cerebrovasculature enabling it to respond to increased neuronal metabolism during times of increased cognitive demand.

### Cognitive Function and Cerebrovascular Responsiveness to Cognitive Stimuli

We observed that AT increased the total cognitive composite score and working memory capacity compared with the control group. We used the total composite score because this is a collective measurement that is more aligned with the demands of daily living. It is also an appropriate measure to use because TCD does not focus on a specific brain region mediating a particular cognitive function. Rarely is only a single individual cognitive domain or subtype used in isolation and the domains are interdependent (Harvey, 2019). However, we also observed an increase in individual cognitive domains, including working memory. This is consistent with previous studies that have reported an increase in individual cognitive domains following 12–24 weeks of AT in older adults who were sedentary (Lautenschlager et al., 2008; Erickson et al., 2011; Anderson-Hanley et al., 2012; Chapman et al., 2013; Guadagni et al., 2020), suffered from mild cognitive impairment (Baker et al., 2010), or had a diagnosis of a dementia (Hoffmann et al., 2016; Sobol et al., 2016). How AT improves cognitive function is not clear, with potential mechanisms being summarized in detail elsewhere (see Bliss et al., 2021). Briefly, those who have reported improvements in cognition have indicated that this may be associated with improved vascular function, cerebral perfusion and/or increased systemic brain-derived neurotrophic factor, which promotes synaptogenesis, neurogenesis and angiogenesis centrally (Lautenschlager et al., 2008; Erickson et al., 2011; Anderson-Hanley et al., 2012; Chapman et al., 2013; Guadagni et al., 2020; Bliss et al., 2021). Acute exercise increases cardiac output, which induces arterial shear stress, promoting endothelial NO synthase expression, which subsequently results in increased NO production (Toda, 2012; Rossman et al., 2018). β-2 adrenoceptor activated endothelial vasodilatation, as occurs in skeletal muscle beds during aerobic exercise, may also induce increased NO production centrally. In any case, NO not only promotes vasodilatation and reduces arterial stiffness, but reduces oxidative stress and inflammation, as well as improving endothelial function and vascular health (Toda, 2012; Rossman et al., 2018). Centrally, this results in improved cerebral perfusion where it also promotes the delivery of systemic molecules, such as BDNF, to the brain, where its effects can be exerted and further promote cognition (Vaynman et al., 2004; Devika and Jaffar Ali, 2013; Sleiman et al., 2016). Therefore, our findings and those from previous studies suggest that improvements in cognition following AT may be due to improved vascular health, which promotes improved cerebral perfusion and CVR during times of increased neuronal metabolism (i.e., improved NVC) (Guadagni et al., 2020). This is supported by the increased cerebrovascular function we also observed.

We observed that following the intervention, the exercise group had a higher total composite CVR to cognitive stimuli than the control group, as well as a higher CVR to each of the individual cognitive stimuli, excluding the Picture Vocabulary Test and the Flanker Inhibitory Control and Attention Test. To our knowledge, we are the first to evaluate the effects of AT in sedentary, obese, older adults who are at risk of cognitive decline. Our findings support those of others who have measured the effects of an AT intervention on NVC and another study which measured differences in NVC between aerobic exercise trained and sedentary older adults. Ozturk et al. (2021) demonstrated that CVR to a working memory task improved following 6 months of AT in young adults with spinal-cord injuries and Fabiani et al. (2014) reported that older adults with greater aerobic fitness had higher NVC capacity. Both studies suggest that improvements in NVC are due to improvements in cardiovascular function, specifically endothelial function. Ozturk et al. (2021) suggested that those suffering from spinal cord injuries have reduced cognitive and endothelial function, which is partly demonstrated by increased arterial stiffness. In other words, a reduction in endothelial function may result from increased arterial stiffness, which reduces the capacity of the cerebrovasculature to supply oxygen and nutrient rich blood to the brain, thus resulting in reduced cognitive function. The authors also suggested that AT reduced arterial stiffness and improved endothelial function in this cohort, thus improving cognition. The characteristics of our participants with metabolic syndrome and those with spinal cord injury are very different. However, one central theme shared among those with spinal cord injuries and those with the metabolic syndrome is that they have reduced endothelial function and increased chronic low-grade systemic inflammation and, therefore, poor cardiometabolic status (Lteif et al., 2005; Mohammadi et al., 2015). Hence, it is clear that improved endothelial function is pivotal in improving cerebrovascular function and cognition. Further, the total composite CVR to cognitive stimuli decreased in the control group. Since this measurement describes NVC and is reflective of the relationship between the brain and its vasculature, this finding also indicates that reduced endothelial function promotes further decline in cerebrovascular and cognitive function and that improving endothelial health is pivotal to improving overall brain health (Duchemin et al., 2012; Toth et al., 2017).

### Correlations Between Exercise Sessions Completed and Cerebrovascular Function and Cognition

The exercise group completed 40 AT sessions on average over the 16 weeks. This equates to approximately 2.5 sessions per week or approximately 100 min of mixed intensity AT per week. This is less than that of the current physical activity guidelines described by both the Australian Department of Health and the American College of Sports Medicine (DOH, 2014; Piercy et al., 2018). Only one other study has deviated from these guidelines and it was reported that 90 min of moderate intensity exercise per week for 12 weeks improved cerebral perfusion and cognition in older, apparently healthy adults (Maass et al., 2014). We observed a very strong positive correlation between the number of exercise sessions completed and the total composite CVR to cognitive stimuli. This finding indicates that a dose-response relationship between these variables may exist and that NVC, a vital regulatory function in cerebrovascular function and neuronal metabolism, can be improved with less than 100 min of AT per week for 16 weeks. There were no significant correlations between the number of exercise sessions completed and the CVR to hypercapnia and total composite cognitive scores. This finding may suggest one of three things: Firstly, some exercise is better than none in improving cognition and CVR to hypercapnia, which reflects the autoregulatory function of the cerebrovasculature. Secondly, a dose-response relationship between these variables exists, but it was too low a dose to be determined in this study. Thirdly, NVC is more sensitive to exercise training than both CVR to hypercapnia and total cognitive capacity. In any case, further studies examining this relationship between the amount of exercise and improvements in overall brain health are warranted because it may be more attainable to promote and prescribe shorter bouts of exercise to improve exercise compliance and engagement in the community, particularly in older adults.

### Methodological Considerations

While we aimed to recruit an even number of men and women, it was not possible due to several confounding factors, such as geographical distance from the study location and potential participants not meeting the inclusion criteria. We acknowledge that the sex differences between the participants of this study may have cofounded our data, even though there were no significant differences between the groups in distribution of men and women. Additionally, Figure 1 reflects the relatively low engagement of the target region (i.e., Ipswich) in health or at least health-related studies. This may have also contributed to the low uptake of the study by men. We noted that there were no significant treatment × intervention interaction effects in any of the data that was collected to define participant characteristics at baseline. This may have been influenced by the fact that the exercise group appeared marginally healthier than the control group at baseline, thus masking potential covariates that may lead to improvements in both cerebrovascular function and cognition. Additionally, the majority of exercise prescription was AT with a small component of resistance training. The resistance exercises were utilized and prescribed in a manner which predominantly focused on the recruitment of the aerobic energy system (i.e., aerobic-resistance training). In any case, we cannot exclude that resistance training may have influenced the results of this study. It must be acknowledged that another potential limitation to this study was that those in the control group may have participated in exercise sessions outside of the study or chose to make lifestyle changes, such as altering their nutritional intake. All included participants agreed at the start of the trial to continue with their current lifestyle and nutritional choices. In an attempt to encourage this, we informed all participants in the control group at Week 0 that once the study concluded that they would be provided with the entire exercise regime, including instructions on how to perform the exercises. We also made regular contact with the control group in an attempt to ensure compliance. Future studies may benefit from having a sham exercise group as a control or another arm added to the study, in which participants do not participate in exercise, rather they interact socially. The latter may be particularly important to control for given that there is evidence that socialization and re-socialization following isolation can improve cognitive performance and brain activity in animals (Rivera et al., 2020) and older adults (Gallucci et al., 2009). We did not use the gold-standard maximal oxygen uptake test to quantify aerobic exercise capacity in this study. Instead, a 6MWT was used, because it is a safe, inexpensive, tolerable and reliable measure of functional exercise capacity commonly utilized in older populations that may also increase compliance for future testing in this population (ATS, 2002; Maniscalco et al., 2006; Ross et al., 2010).

## Conclusion

In conclusion, 16 weeks of AT increased the CVR to hypercapnia, CVR to cognitive stimuli and total composite cognitive score in sedentary, obese, older adults compared with the control group. A very strong relationship was observed between the number of exercise sessions completed and CVR to cognitive stimuli, but not for CVR to hypercapnia or total composite cognitive score. These results indicate that cognition and the responsiveness of the cerebrovasculature to physiological stimuli can be improved by as little as 100 min of exercise per week in previously sedentary, older, obese adults with metabolic syndrome. These individuals are at an increased risk of developing cognitive impairment and, potentially, a neurodegenerative disease such as dementia. Future studies should ascertain the minimum dose of AT needed to improve total cognitive capacity and CVR to hypercapnia in a cohort of older adults.

## Data Availability Statement

The original contributions presented in the study are included in the article/supplementary material, further inquiries can be directed to the corresponding author/s.

## Ethics Statement

The studies involving human participants were reviewed and approved by the University of Southern Queensland Human Research Ethics Committee. The patients/participants provided their written informed consent to participate in this study.

## Author Contributions

EB, RW, PH, and DM conceptualized, designed the study protocol and experiments, and contributed to revisions of intellectual content. EB and DM designed the training protocol for the study. EB collected the data and analyzed the data. EB and DM performed statistical analysis, with all authors contributing to data interpretation. All authors approved the final manuscript.

## Conflict of Interest

The authors declare that the research was conducted in the absence of any commercial or financial relationships that could be construed as a potential conflict of interest.

## Publisher’s Note

All claims expressed in this article are solely those of the authors and do not necessarily represent those of their affiliated organizations, or those of the publisher, the editors and the reviewers. Any product that may be evaluated in this article, or claim that may be made by its manufacturer, is not guaranteed or endorsed by the publisher.
